# To Be Supported, or Not to Be: Images of Older People in Policy and the Reality in Local Communities in Japan

**DOI:** 10.3389/fsoc.2019.00016

**Published:** 2019-03-12

**Authors:** Ken Aoo, Noriko Abe, Mitsunobu R. Kano

**Affiliations:** ^1^Graduate School of Interdisciplinary Science and Engineering in Health Systems, Okayama University, Okayama, Japan; ^2^Research Institute for Local Community, Okayama, Japan

**Keywords:** social innovation, local community, older people, welfare society, social service

## Abstract

Social innovation is not only about tangible new products, services, policies, and laws, but also about changes in societal perceptions, values, and norms. In Japan, current policies for older people, including Long-Term Care Insurance, tend to focus on medical and long-term care and other forms of “support” for older adults such as a pension. Naturally, these policies depict older adults as the “beneficiaries,” or the ones in need of support. However, when we look back at pre-modern Japan, it was not always like that. Although older adults did depend on support from family and community members, they also played an active role as a laborer and caretaker as well as providing useful knowledge for their family and community. Moreover, currently, in different areas suffering from a sharp decline in population, such as Okayama prefecture in western Japan, older people are actually playing the role of the supporter for groups of people who are in need, not only the aged population but also other demographics including young children and parents. Based on this historic “tradition” and the present reality, this paper argues that we need to reestablish the image of (at least some) older people as capable of taking a more active and responsible role in society, and position them as such in the new “welfare society” systems in order to replace the conventional “welfare state” model.

## Background: Social Innovation and Aging

Since the 1990s, social innovation (hereafter “SI”) has attracted the attention of both policy-makers and academics in different regions, including Europe, North America, and Asia (Aoo, 2018). This paper defines SI, in line with the recent literature mainly developed in the United Kingdom and Europe, as:

- A new product, service, or initiative that can solve a social issue and/or create social value in a better way than existing methods, and;
- A process that leads to a change in current perspectives, values, norms, attitudes, relationships, and systems, and finally to the systemic change of the society (Murray et al., 2010; Nicholls et al., 2015).

Although this aspect is often overlooked, SI does not only refer to tangible devices and services, or the enacting of new laws and regulations. Another aspect of SI is serving as a catalyst for changes in the cultural-cognitive perspectives and social norms that are framed by the cultural structure of the society (Bourdieu, 1990; Heiskala, 2007; Klimczuk, 2015).

Aging is both a major social issue that needs to be solved by SI, and a structural change that calls for SI in each society. Especially in the developed world, the classic “welfare state” model (Esping-Andersen, 1990) is no longer sustainable due to slowing economic growth and an aging society, and SI is expected to provide the inclusive framework of a “welfare society” to replace it (Mulgan, 2012).

Still, older people are mostly treated in SI literature as the “beneficiary” or the “target” of social and medical care (Heinze and Naegele, 2012; Heals and Green, 2016). There is no question about the significance of establishing effective measures and support mechanisms for older adults. Nonetheless, this paper argues that (some) older people should be re-recognized as a “supporter” of aging societies, replacing the conventional view that older adults are only a target for support, by using Japan as an example. The argument is based on findings from historical studies that demonstrate that the one-sided view of seeing older people solely as “beneficiaries” is quite a recent phenomenon and not a persistent tradition. This study was carried out by the recommendations of the Okayama University Research Ethics Guidelines, and according to the current Guidelines and national regulations, do not require approval from an Ethics Committee. The study has obtained written informed consent from all participants.

This article takes the following structure. Following the introduction, section The Historical Image of Older People in Pre-Modern Japan introduces a few examples of images of older people in pre-modern Japan which shows them not only as recipients of support. Next, in section “Beneficiarization”: The Shift in the Image of Older People in the Modern Era, it presents how older adults were re-defined in the discourse of modern Japanese society, especially in relation to the establishment of the Long-Term Care Insurance System in 2000. Section (Re-)Changing Reality in Local Communities describes how, mainly based on the example of Okayama prefecture, older people are shouldering the responsibility of supporting others within the community Finally, section Conclusion claims in conclusion that it is not unreasonable to ask for the contribution of older people who are both capable and willing, and is in fact a “re-invention” of a longstanding tradition that will also create a positive economic impact on society.

## The Historical Image of Older People in Pre-Modern Japan

According to the recent findings of historical studies and literature on historical sociology, some form of charitable support for older people has existed in Japanese society since ancient times, based on Confucian values (Ikeda, 1986)^1^. In the Chinese-influenced legal codes of the eighth century, people over 60 years old were entitled to a decrease in taxes and other obligations. Older adults without a spouse were also entitled to receive some support (Shinmura, 1991). Meanwhile, in the medieval and early modern age under *Samurai* (warrior class) rule, it was a common norm in society that the rulers were responsible for helping people in need, including older people without living family members (Ikeda, 1986).

Of course, it is not realistic to believe that such support was continuously provided, and usually, the lords and administrators held children, extended family members, or the neighboring community responsible for supporting older adults, based on filial piety (Takagi, 2006)^2^. Therefore, the family was the primary source of support for older people^3^. On the other hand, older people also supported their families in various ways, from assisting with agricultural, and domestic work to taking care of and educating children and the sick. They were respected in the local community for their knowledge and contributions to familial or community functions and ceremonies and given a higher rank in public meetings (Shinmura, 1991). There is a famous folktale called “*Ubasute* (abandoning old women)” about an old woman who was kept hidden by her son while the ruler told his subjects to abandon “unproductive” old people. The advice she gave her son in response to the lord's impossible demands (e.g., to make a rope out of ashes) finally convinced the ruler of the usefulness of the wisdom of older adults (Kawai, 2017).

## “Beneficiarization”: The Shift in the Image of Older People in the Modern Era

After the late nineteenth century, as the population began moving to big cities from their hometowns and the structure of industry changed, older people lost the roles they held in traditional communities. The modern Japanese government also held children responsible for the support of older adults in the Civil Code (Shinmura, 1991), apart from philanthropic support provided through foundations with royal patronage (Yamaoka, 1993).

After defeat in WWII, the so-called “Japanese-Style Welfare Society” took form, consisting of weak public support^4^, welfare and benefits provided by employers, and gender-based division of labor (positioning women as the caretakers in the family). This system persisted until the 1980s (Ueno, 2011; Aoo, 2017).

Since the 1970s, the issues of bedridden older adults and so-called “social hospitalization,” which kept the elderly in hospitals that functioned as practical nursing homes, started to be recognized as a major problem in Japanese society (Aoo, 2017). At the same time, the “Japanese-Style Welfare Society” began to dissolve after the 1990s due to slowing economic growth and changing employment and familial systems, such as the increase in temporary and part-time employment, and a reduction of the benefits provided by corporations (Tanaka, 2017).

The Long-Term Care Insurance System (LTCI) was launched in 2000 to respond to a rapidly aging society. The LTCI system itself was a landmark example of social innovation which created a new care services industry for older adults and achieved (at least partly) the goal of “socializing” the caretaking of older adults, that is, of society shouldering the burden together with family members^5^. On the other hand, LTCI defined older people as “recipients” of care services, and it created a major change in people's mindsets in a fairly short period, as the norm shifted from family-based care to services-based care^6^. Authors such as Miyamoto (2014) have emphasized the importance of empowering older people, utilizing their capabilities, and creating spaces for them to contribute to society. But in the policy arena, even with a major effort by the government to introduce communal and volunteer services under the banner of “Community-Based Integrated Care,” these concepts remained marginal (Aoo, 2017). Tsutomu Hotta of Sawayaka Fukushi Foundation, who was involved in older adults care policies, recalls two of Japan's failures as (i) excluding older adults from the job market uniformly by their age; and (ii) not being able to provide enough non-profit organizations to invite older people to participate in activities to support their communities (Hotta, 2008).

## (Re-)Changing Reality in Local Communities

However, the reality in local communities, especially in rural areas where populations are shrinking and local governments are being stretched thin by public sector reforms and merging municipalities, is necessitating older people to take on a new role. The northern area of Okayama prefecture in western Japan, where the authors reside, suffers from an aging population and depopulation. In Tsuyama, a town in the area, older people (above 60) are visiting other older adults and assisting with housework for a small fee, utilizing neighborhood association networks. They also provide support to young parents and have become organizers of community events. Ueyama, another mountainous area in northern Okayama, relies on mutual support networks of older people to provide welfare and social services^7^.

Similar mutual support networks in the area are now being developed, based on neighborhood associations or school districts, including in the towns of Misaki, Kume-Nan, and Kagamino and Maniwa. There are active inter-area visiting and mutual learning among communities of older people. When the authors interviewed local supporters in Kume-nan town who run communal spaces and provide assistance for the livelihoods of others, some of them say “I did not have many ties with my local community when I was young because of my work, but now that I've retired, I want to help the community” or “We need to create an environment to help each other before we become the ones who need others' help.” Requests and inquiries from local leaders such as the heads of neighborhood associations wishing to learn about such mutual support mechanisms are growing day by day. In the interviews, Mr. Kokumai of Smile Chiwa, a non-profit in Tsuyama city, also called for the participation of community members by saying “There are many people living alone without family, so if we consider them to be like a member of our own family, then we should do what we can.” These initiatives and networks are starting to spread in urban areas as well, not only in isolated rural areas where the aging of the local population has become a fact.

From these changes, we can see older people defining new roles for themselves in aging communities and finding their *raison d'être* there. This is not only happening in Okayama, and according to Yomiuri Shimbun, a major national newspaper, many areas are having difficulty finding welfare commissioners to monitor people in need in local communities, and around 20% of municipalities have to raise (or abolish) their age limit for commissioners, which used to be under 75 years old^8^.

## Conclusion

This article has presented the changing image of older people over history and showed how older adults are coming back again as supporters of local communities.

One thing which should be made clear is that this paper is not describing this situation to absolve governments of their responsibilities by (over-)stressing the roles of family and community members, as some types of discourse on the “re-invention of tradition” tend to do. Contrarily, what we see as a major change/SI needed in Japanese society is a shift away from the dichotomy of full-time workers and the unemployed to a more diverse model of employment status (see Table 1). Especially after WWII, the mainstream model in place in modern Japanese society was either being employed full-time (often for life, until retirement age) or not being employed at all (including homemakers)^9^. Perhaps such a homogeneous workforce was useful in the era of industrial development with the accompanying large population of young people. However, in the age of an aging and shrinking population and a post-industrial society, we propose that these active older people, among other informal supporters, be given suitable value and rewards within the regimes of social welfare and services, including connections with others, recognition, and financial remuneration even for their part-time contributions. Such an approach should not only help to encourage more people to participate in mutual support activities and to increase the well-being of the community, but also to create more economic and social values as a part of the “solution economy” or “care economy,” or more service-oriented economies (Heinze and Naegele, 2012; Sgaragli and Giacomo Brodolini Foundation, 2014).

**Table 1:** Model of changing workforce systems.

**Post-WWII Model**	**Post-Industrial Model**
- The dichotomy of paid full-time workers and unemployed	- More diversified employment types and payments
- Domestic caretakers and local volunteers were unpaid, often divided by gender roles	- Caretakers are rewarded according to their contributions

Perhaps it is useful to be aware of possible “negative” side effects, including the presence of older generations staying “active” potentially keeping the younger generations locked into a subordinate position in societies and cultures whose concept of authority is based on a seniority system. There is a need to formulate more flexible working patterns for older people. Still, it seems that there is a lot we can learn from our own premodern history that will help us develop possible new “welfare society” concepts that include active roles for older adults, such as the word “*Inkyo Shigoto*,” which means “a job after retirement” (Ogasawara, 2012).

Changing the image of older people from a mere “beneficiary” of social services to a more active and influential party can lead to changing societal values and norms and can be the basis of developing new SI initiatives and systems. Starting from the argument presented by this brief paper, both academics and practitioners should work together to gather more evidence and knowledge from the society, and to propose new policy systems. Then each society may need to consider what the most appropriate image and perception of older people (together with other groups) would best fit the future social innovation needed.

## Author Contributions

KA conducted a research on social innovation theories and concepts, historical analysis, and a part of present day situation in local communities. NA added details and observations from the local communities. MK supervised the conceptual framework and added descriptions to clarify the framework.

### Conflict of Interest Statement

The authors declare that the research was conducted in the absence of any commercial or financial relationships that could be construed as a potential conflict of interest.

## Footnotes

^1^Values and the practice of filial piety, which is based on the Confucian literature of China, were widely accepted in East and Southeast Asian societies including China, the Korean peninsula, Vietnam, and Japan, and still remain a major factor in determining the behavior of children toward their parents (Chow, 2004).

^2^Mutual support systems in pre-modern Japan called *Ko* or *Yui* were major safety nets for ordinary people, but it was not easy for older adults without a regular cash income to pay premiums for the systems (Najita, 2009). Older people without family sometimes had to be hired by a wealthier family or live a vagrant life (Takagi, 2006).

^3^Still, family support was not unconditional and was often subject to negotiation (Takagi, 2006).

^4^Old people's homes run by the government only accepted poor older adults without living family members, and it also created a stigma of receiving public welfare services (Ueno, 2011).

^5^Please see (Aoo, 2017) for the details of the LTCI system and its issues, including the sustainability of financial and human resources.

^6^According to research conducted by the Ministry of Health, Labor and Welfare in 2016 (page 24, Figure 2-2-27), only 18.6 percent of respondents say that they want to “stay home, taken care of mainly by family members” when they become frail, and others showed their preference to not depend on care by family members, including “stay at home without depending on family (37.4 percent),” “stay at home with both family care and external care services (17.5 percent),” “live in care facilities or medical institutions (total 25 percent).” The MoHLW website: *https://www.mhlw.go.jp/file/04-Houdouhappyou-12601000-Seisakutoukatsukan-Sanjikanshitsu_Shakaihoshoutantou/001_2.pdf* (Accessed 30th Dec, 2018, in Japanese)

^7^Research Institute for Local Community website: *http://www.npominken.ip/minmobi/* (Accessed 12th Oct, 2018, in Japanese)

^8^Yomiuri Shimbun 8th July 2018 “The Aging of Welfare Commissioners Getting Serious: Many forced to relax the age limit of under seventy-five.” According to the article, the average age of welfare commissioners is 66 years old.

^9^Of course, there was a large number of self-employed businesses and farmers during this time.
